# Controlling Neglected Tropical Diseases (NTDs) in Haiti: Implementation Strategies and Evidence of Their Success

**DOI:** 10.1371/journal.pntd.0004954

**Published:** 2016-10-05

**Authors:** Jean Frantz Lemoine, Anne Marie Desormeaux, Franck Monestime, Carl Renad Fayette, Luccene Desir, Abdel Nasser Direny, Sarah Carciunoiu, Lior Miller, Alaine Knipes, Patrick Lammie, Penelope Smith, Melissa Stockton, Lily Trofimovich, Kalpana Bhandari, Richard Reithinger, Kathryn Crowley, Eric Ottesen, Margaret Baker

**Affiliations:** 1 Ministry of Public Health and Population, Port-au-Prince, Haiti; 2 IMA World Health, Port-au-Prince, Haiti; 3 Hôpital Sainte Croix, Léogâne, Haiti; 4 University of Notre Dame, Léogâne, Haiti; 5 RTI International, Washington, District of Columbia, United States of America; 6 IMA World Health, Washington, District of Columbia, United States of America; 7 Centers for Disease Control and Prevention, Atlanta, Georgia, United States of America; 8 U.S. Agency for International Development, Washington, District of Columbia, United States of America; 9 RTI International Consultancy, Washington, District of Columbia, United States of America; Common Heritage Foundation, NIGERIA

## Abstract

Lymphatic filariasis (LF) and soil-transmitted helminths (STH) have been targeted since 2000 in Haiti, with a strong mass drug administration (MDA) program led by the Ministry of Public Health and Population and its collaborating international partners. By 2012, Haiti’s neglected tropical disease (NTD) program had reached full national scale, and with such consistently good epidemiological coverage that it is now able to stop treatment for LF throughout almost all of the country. Essential to this success have been in the detail of how MDAs were implemented. These key programmatic elements included ensuring strong community awareness through an evidence-based, multi-channel communication and education campaign facilitated by voluntary drug distributors; strengthening community trust of the drug distributors by ensuring that respected community members were recruited and received appropriate training, supervision, identification, and motivation; enforcing a “directly observed treatment” strategy; providing easy access to treatment though numerous distribution posts and a strong drug supply chain; and ensuring quality data collection that was used to guide and inform MDA strategies. The evidence that these strategies were effective lies in both the high treatment coverage obtained– 100% geographical coverage reached in 2012, with almost all districts consistently achieving well above the epidemiological coverage targets of 65% for LF and 75% for STH—and the significant reduction in burden of infection– 45 communes having reached the target threshold for stopping treatment for LF. By taking advantage of sustained international financial and technical support, especially during the past eight years, Haiti’s very successful MDA campaign resulted in steady progress toward LF elimination and development of a strong foundation for ongoing STH control. These efforts, as described, have not only helped establish the global portfolio of “best practices” for NTD control but also are poised to help solve two of the most important future NTD challenges—how to maintain control of STH infections after the community-based LF “treatment platform” ceases and how to ensure appropriate morbidity management for patients currently suffering from lymphatic filarial disease.

## Author Summary

We present evidence of Haiti’s successful neglected tropical disease (NTD) program targeting lymphatic filariasis and soil-transmitted helminths and the methods used to achieve this success. By 2012, Haiti’s NTD program had reached full national scale, with such consistently good treatment coverage that the program is now able to stop treatment for lymphatic filariasis in much of the country. These findings are in line with the predictions and expectations of the global community for countries where high coverage is achieved for program implementation. In addition to the evidence of successful program outcomes, we present a detailed description of how the program was implemented—from facilitating the effectiveness of the drug distributors to improving drug logistics and supporting a well-informed population. These methods described can be used to inform the design of other mass drug administration programs and enhance the development of global “best practices” guidance.

## Introduction

Neglected tropical diseases (NTDs) are a group of 17 parasitic, bacterial, and viral infections affecting more than 1 billion people globally [1]. Seven of these are known as the preventative chemotherapy NTDs because they can be eliminated or controlled by administering medicines to entire eligible populations or large segments of these populations in an effort to reduce transmission of infection and prevent disease.

Four preventative chemotherapy NTDs are endemic in Haiti—lymphatic filariasis (LF) and the three soil-transmitted helminth (STH) infections caused by *Ascaris*, *Trichuris*, and hookworm. LF is one of the world’s most debilitating parasitic diseases, causing lymphedema, elephantiasis, hydrocele (enlarged scrotum by fluid accumulation), and hidden internal damage to the lymphatic and renal systems of affected individuals [2]. Furthermore, as a disease associated with stigma, despair, hopelessness, embarrassment, ridicule, frustration, and economic burden [3], LF can also cause significant mental health complications that reach far beyond even its physical morbidity [4]. STH causes a wide range of intestinal symptoms and has also been associated with poor cognitive development and learning capacities in children [2]. Furthermore, STH can cause anemia in women of child-bearing age, which is both detrimental to the mother’s health during pregnancy and can lead to low birth weight [5].

Haiti is one of only four countries in the Americas where LF transmission still occurs, and it is home to the largest at-risk population in the region [6,7]. In Haiti, LF is caused by *Wuchereria bancrofti* filarial parasites, primarily transmitted by *Culex quinquefasciatus* mosquitoes [7,8]. In 2000, a nationwide mapping exercise reported an infection prevalence (assessed by filarial antigen) as high as 45% in children 6 to 11 years old, with infected children identified in 117 of the country’s present 140 communes (133 communes at that time) [9]. Nationwide “mapping” for STH infections was carried out in 2002, with all of Haiti’s ten departments (previously reported as nine due to redistricting) reporting >20% prevalence and two of these with >50% prevalence [10].

Addressing such NTD challenges is complex. Although health experts and international organizations have presented general recommended approaches for integrated programs targeting NTD control or elimination [11], the global portfolio of “best practices” can only grow through the addition of detailed records of individual national experiences. Indeed, the success of national NTD programs (or at a minimum, their avoidance of failure) depends on understanding the *methods* used to implement and assess programs in those countries where NTD programs are already mature or complete. In this regard, Haiti could make important contributions to the accumulating experiences of implementing integrated NTD programs [12,13]. This current report, while recognizing the importance of the contributions of all of Haiti’s principal NTD stakeholders[14], focuses on the efforts implemented by the Haitian government and a large project (ENVISION) supported by the U.S. Agency for International Development (USAID). ENVISION is led by RTI International and is being implemented in 19 countries globally [http://www.ntdenvision.org]; in Haiti, IMA World Health leads the ENVISION activities.

## Methods

### Context

#### Socio-geographic context

Haiti, situated adjacent to the Dominican Republic on the island of Hispaniola, has a population in excess of 10 million people and is a low-income country, with 59% of its citizens living below the poverty line. It has an under-five mortality of 76 per 1,000 live births, and only 48% of its population has access to an improved water source [14,15]. The entire population of Haiti lives in areas at risk for LF and STH.

Haiti is divided into three administrative tiers—national level, departments (equivalent to regions in other countries), and communes (equivalent to districts); the commune is the programmatic implementing unit for all health programming, including NTD efforts. Approximately 60% of the population lives in rural areas, and 40% are spread across four major urban areas, the largest being Port-au-Prince (population of 2.5 million). The geographic areas where mass drug administration (MDA) was supported by USAID’s ENVISION project (the focus of this paper) were mostly in semi-urban and urban communities.

In the past decade, Haiti has faced a myriad of development challenges, including political crises, hurricanes, a devastating earthquake, and a deadly cholera outbreak in the aftermath of the earthquake [16].

#### Background of the NTD program in Haiti

The national LF and STH programs were developed independently; however, the two programs integrated their MDA activities in 2008.

The national program to eliminate LF (NPELF) in Haiti was established in 2001, with the objectives of eliminating transmission of LF and reducing the suffering of persons with the clinical and chronic manifestations of LF disease. Under the leadership of the Ministry of Public Health and Population (MSPP), the NPELF overcame many challenges (including the political crisis that began in 2013 and escalated into a state of high-level insecurity, with shootings and kidnappings that directly affected program staff; interruption in financial support; and the 2010 earthquake) to successfully scale up the World Health Organization (WHO)-recommended MDA strategy, reaching at least 65% of the population once yearly with two medicines– 400 mg of albendazole (ALB) and an age-based dose of diethylcarbamazine (DEC) [16–19].

Efforts to control STH focus on maintaining low prevalence and intensity of infection, and are coordinated by the MSPP and the Ministry of Education (MENFP). As per WHO strategy, Haiti’s approach to STH control is based on periodic treatment of at-risk populations (i.e., pre-school and school-aged children, women of reproductive age) living in endemic areas with an anthelminthic, typically ALB or mebendazole. In areas where STH prevalence is greater than 50% or between 20% and 50% of the surveyed population, twice-yearly and once-yearly treatment is recommended, respectively [2]. In addition, WHO recommends education on health and hygiene and—where possible—provision of adequate sanitation. In 2000, the MSPP set up a program to focus on wide-scale delivery of anthelminthic drugs, and since 2003, the MENFP has additionally used its school health program to focus on promoting hygiene and sanitation at schools.

**Fig 1:**
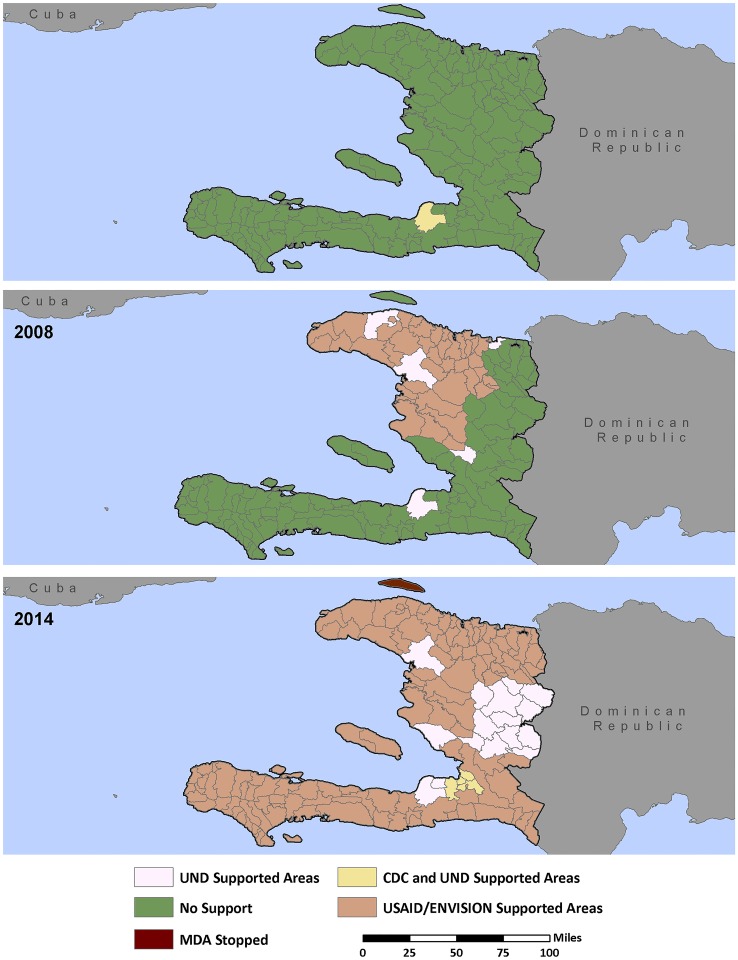
Geographic regions of partner support for the National NTD Program (2000–2014). Data Source: USAID’s NTD Database. Disclaimer: Data may have not yet been approved by at least one level in the USAID NTD data review process.

**Fig 2:**
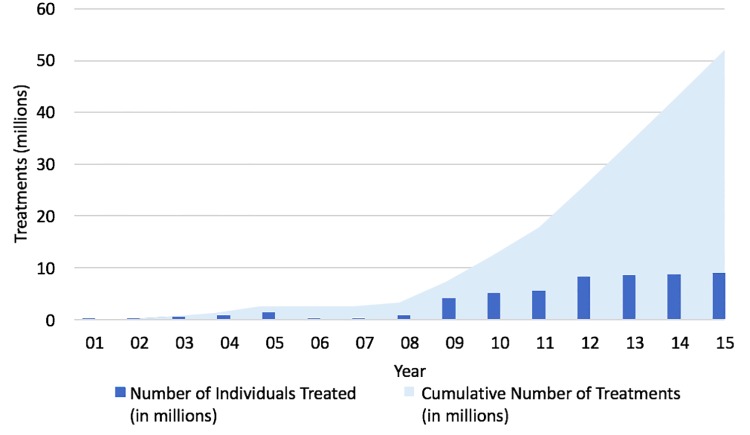
Number of people treated and cumulative number of treatments given by program year.

In 2008 Haiti formed a single national NTD program—Le Projet des Maladies Tropicales Negligées (MTN)–to consolidate their STH and LF programs. Because STH programs are often integrated with LF MDA in areas where the diseases are co-endemic and ALB is a mainstay of effective treatment for both [20], the national government thought it prudent to unify the efforts of these independent disease programs and government departments. In addition to the benefits of combining two programs, the Haitian NTD program was further bolstered in 2008 when it started receiving external funding support through USAID. The resulting partnership, led by the MSPP, included USAID and its implementing partners—IMA World Health and RTI International under the ENVISION project, the U.S. Centers for Disease Control and Prevention (CDC), and the University of Notre Dame. Despite a slow start during the first eight years of the LF program, treatment numbers rapidly increased after 2008 when stable funding became assured for much of the country (Figs 1 and 2). By 2012, full geographic coverage of all 140 endemic communes had been achieved, and by 2014, a limited number (20) of communes had satisfied the criteria to stop MDA for LF–marking the start of the surveillance phase of the LF elimination program. Because MDA for LF is community-wide, those individuals treated with DEC and ALB also include school aged children and women of reproductive age—two populations also at risk for STH infection.

### MDA Program Implementation

#### Human resources

The NTD program is led by the government and managed with the support of ENVISION. Human resources comprise a team of health professionals within the MSPP’s units for LF and STH (at national, department, and commune levels), school inspectors employed under the MENPF, and IMA staff. At the community level, a cadre of community volunteers (22,654 volunteers in 2014), selected especially to work on the NTD program, ensure that the medicines are distributed to the target population. This cadre is composed of community drug distributors (CDDs) supported by promoters, who in turn are supported and chosen by community leaders working with the MSPP.

CDDs can be health workers, students, teachers, and other individuals chosen from within the community. They are selected based on several criteria: they must live in the community where they will distribute the medicines, be well known and respected by the population, and be able to follow instructions given during MDA training. Because CDDs live in and know the community within their distribution-post areas, they are able to identify easily, and follow-up directly, any individuals who have missed MDA. Each CDD is expected to treat approximately 300 persons during each MDA. Promoters, including health workers, teachers, and political leaders, are chosen based on having had experience working with the health center in previous health campaigns (e.g., immunization) and having strong standing in the community. As well as recruiting, training, and supervising CDDs and promoters, the community leaders’ role is to sensitize the population at community meetings, schools, churches, and other venues; to meet with and inform other political and church leaders who have influence in the community; and to deliver MDA kits to distribution posts and help manage stock. The leaders report to the MSPP staff member managing the local health post.

CDD turnover is low (less than 10% per year), and the NTD program does not encourage changes. Incentives paid (often less than other programs) range from US$25 to US$65 per month worked, for up to three months, and are settled at the end of the MDA. The main source of motivation is believed to be the high level of engagement of national-level program managers who interact directly with CDDs, continually listening to, encouraging, and motivating these CDDs—by letting them know the importance of their work and that it is seen and appreciated. However, further qualitative studies are needed to gain a deeper understanding of what motivates the drug distributors.

#### Social mobilization and information, education, and communication

In Haiti, the MDA medicines, DEC and ALB, are distributed to communities at fixed treatment posts, including schools, set up throughout the community. The entire community is invited to visit the posts; schoolchildren are treated at their schools. The information, education and communication (IEC) efforts are multi-channeled, including posters, flyers, banners, sound trucks, radio, television spots, and community meetings held in schools, churches, and markets. Program managers also hold live press conferences on local radio stations, which include answering listener call-in questions. Another factor contributing to the high level of community awareness of the MDA was the program’s campaign style: for a short period of time, the streets were flooded with volunteers and staff, who were made highly visible by the specially designed NTD program tee-shirts they wore. Messages focused on how to get treatment and on side effects.

The IEC approach was informed by results of knowledge, attitudes, and practices (KAP) surveys conducted in 2012 and 2013, which asked how people hear about the MDA program. As a result of survey information, a higher proportion of funding was spent on banners, fliers, and radio and television messages, and a lower proportion on posters. Importance was also placed on messaging through megaphones mounted on trucks.

#### Directly observed treatment

The Haiti NTD program policy is for all treatment to be directly observed. Training emphasizes watching each participant in the MDA take the medicine, and the policy is enforced by supervisors. If people request medicines to take home with them, they are to be refused. If a person arrives at the treatment site and has not yet eaten, the CDD requests that the person eat first and then return for treatment.

#### Handling adverse events

Because even minor side effects can negatively affect treatment coverage, providing clear information on managing side effects to the community is important. CDDs were trained to encourage people to eat before treatment in order to reduce side effects.Information on potential side effects in included as a key element of all training and social mobilization efforts. Data reported previously from Léogâne showed that although the percentage of respondents who reported any side effects within a day of MDA was approximately 25% (with nausea being the most common symptom reported), the percent of the population reporting *fear* of side-effects was <2%. Such findings were similar to those reported in other studies in Haiti following multiple rounds of MDA, where the most common symptoms were headache (36%) and gastrointestinal complaints (28%) [21]. No serious adverse events have been reported during implementation of the nationally supported efforts so far.

#### Training

Essential to success has been the large emphasis on training—a cumulative total of 102,375 persons (medical personnel, community leaders, community promoters, and CDDs) were trained from 2009 through 2014. Given the low turnover of CDDs, a two-day training is provided in each commune prior to its first MDA round, with shorter refresher trainings (of one day) provided in following years. Key topics include information on diseases and reporting, followed by an MDA simulation. In addition, the ENVISION finance team trains the MSPP’s departmental accounting team annually in a one-day session.

**Fig 3:**
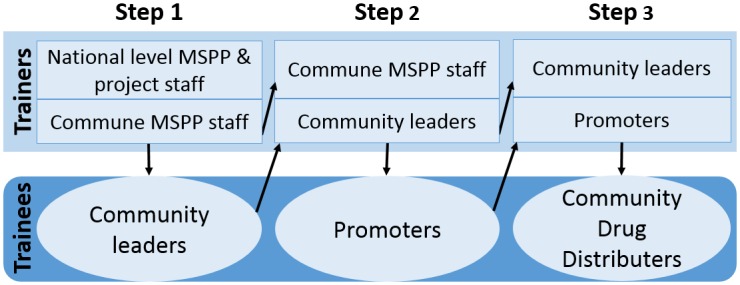
Cascade training.

ENVISION’s training approach avoids dilution of content—the often-cited potential weakness associated with the cascade training-of-trainers model—by ensuring overlap in the layers within the cascade: community leaders are trained by a joint team of national, departmental, and commune-level MSPP staff; community leaders are then joined by commune-level staff to train the promoters, who in turn are supported by the community leaders and district staff to train drug distributors (Fig 3). This overlapping cascade model ensures that trainers continue to be accompanied and supported by their trainers for a period of time before they become senior trainers.

#### Supervision

Supervisory oversight is provided not only at community level, but also at district and national levels. The CDDs are supervised by a promoter, with each promoter supervising an average of nine CDDs. Supervisor responsibilities include checking that the correct number of CDDs are in place; ensuring that distribution posts are appropriately located and that they have the necessary supplies; and reviewing accuracy of completed data registers. Supervision at the community level is conducted jointly by ENVISION and staff from both the MSPP and the MENPF. Following the MDA, a post-event review is held at the local level, where MSPP staff and the community CDDs review program data before reporting, reflect on lessons learned, and strategize on how to improve coverage in future years.

#### Drug supply chain

Underlying the program’s drug distribution effort is a strong supply chain. Drugs are received in the country by the national medical stores and then transferred to ENVISION for storage, repackaged by pharmacists, and distributed to communes two to three days in advance of the MDA. During 2009 to 2014, there were no reported instances of drug stock-outs during MDA activities. Furthermore, records consistently have demonstrated a very low percent of drug loss each year—of a total of 136 million DEC tablets and 27 million ALB tablets received from 2009 to 2014, only 0.2% of them were lost.

#### Program monitoring and evaluation

The NTD program in Haiti has been guided by information collected through the ENVISION monitoring and evaluation (M&E) framework, designed according to WHO guidelines [22,23]. The program routinely captures data on all persons treated and on the drugs used—as recorded by CDDs during MDA rounds and reported to national level through the health system. Data are currently stored by ENVISION, and the MSPP is in the process of setting up the standardized WHO Integrated NTD Database.

Key indicators—assessed at commune and national levels—include *process* indicators that measure fidelity between program implementation and program plans, *outcome* indicators, and *impact* indicators that measure infection to identify communes ready to stop MDA. Coverage is the key outcome-level indicator and is defined as follows:

- Program coverage: number population ingesting medicines/ eligible population targeted for treatment;
- Epidemiological coverage: number population ingesting medicines / total population areas where program is implemented;
- National coverage: number population ingesting medicines / total population living in all areas where PC is required; and
- Geographical coverage: number of communes having received MDA / all communes that required MDA

Other data are collected through a number of special evaluations. Post-treatment coverage surveys, involving 30 clusters each, were conducted, with data collection support provided by CDC following WHO LF monitoring guidelines [22]. These surveys were carried out in selected communes in 2012 and 2013 and included responses from a total of 5,412 and 10,907 persons from six and seven communes respectively, with more in-depth KAP information obtained from a sub-sample of 1,241 persons (in 2012) and 3,577 persons (in 2013).

Following WHO guidelines, sentinel site and spot-check sites are used periodically to measure impact on infection rates, and transmission assessment surveys (TAS) are used to determine whether MDA can be stopped [24]. The MSPP also conducted national STH surveys in 2005 and 2013.

## Results

### Effectiveness in Reaching the People—Program Coverage

Treatment coverage is a measure of whether programs are on track to achieve program goals and control/elimination targets. Good coverage was reported (Table 1)– 100% geographical coverage was achieved in 2012 and national coverage targets were reached for both STH (≥75%) and LF (≥65%) across most communes. In communes supported by ENVISION, the average epidemiological coverage for LF MDA rounds ranged from 86% to 94% (Table 1). The number of communes with coverage below the 65% target ranged from one to five in any given year, with no systematically low-performing communes identified.

**Table 1:** Treatment coverage in Haiti, 2009–2014.

	2009	2010	2011	2012	2013	2014
National LF Program*						
Geographical coverage	-	64.2%	100.0%	100.0%	80.7%**	71.0%**
National coverage—LF (ALB+DEC)	35.4%	42.9%	90.3%	75.2%	64.0%**	48.1%**
# persons treated (nationwide)	3,058,566	3,947,635	8,789,048	8,071,399	7,047,600**	5,469,024 **
# persons treated (with ENVISION support)	2,111,826	3,599,143	4,784,104	4,848,373	4,998,704	5,159,277
# communes where infection threshold reached and MDA stopped	0	0	0	0	0	47
National STH Program*						
National STH coverage (ALB+DEC integrated MDA)	30.8%	42.0%	89.4%	79.9%	71.8%	Unavailable
National STH coverage: (second round ALB only)	59.1%			58.5%	15.5%	
Epidemiological Coverage in ENVISION-supported Areas						
# districts supported	46	76	106	106	106	97
Average epi. coverage	86.2%	94.2%	93.9%	89.7%	90.1%	90.0%
# communes with <65% epi. coverage	3	2	1	5	1	1

*Taken from WHO NTD Database: http://www.who.int/neglected_diseases/preventive_chemotherapy/lf/en/

**Reduced numbers over the years reflect the fact that treatment has been stopped in an increasing number of communes.

Post-treatment coverage surveys were used to validate the coverage routinely reported by CDDs. Results of the coverage surveys generally confirmed that coverage rates were at least 65%, and often substantially higher (Table 2).

**Table 2:** Results of surveys conducted in 2012 and 2013 to assess treatment coverage rates.

Region/commune (Year)	Reported epidemiological (at risk) coverage	Survey sample size	Survey epidemiological (at risk) coverage	Design effect^1^
**2012**				
Northwest/Baie-de-Henne (2012)	69.7%	866	69.6% (61.0%–78.0%)	7.1
Artibonite/Desdunes (2012)	82.6%	988	82.7% (76.0%–89.0%)	6.3
Artibonite/Gros-Morne (2012)	82.1%	728	86.0% (80.0%–92.0%)	5.7
Northwest/Jean-Rabel (2012)	87.2%	926	90.4% (87.0%–93.0%)	2.2
North/Limbé (2012)	87.5%	902	91.7% 89.0%–95.0%	2.5
North/Pignon (2012)	80.9%	1,002	90.7% (88.0%–93.0%)	2.1
**Average/total**	**81.7%**	**5,412**		
**2013**				
South/Aquin (2013)	102.0%	1,913	87.0% (82.0%–92.0%)	5.4
Southeast/Cayes-Jacmel (2013)	102.45%	2,354	90.7% (88.0%–94.0%)	5.3
Southeast/Jacmel (2013)	83.8%	2,134	80.2% (74.0%–86.0%)	5.4
Nippes/L’Asile (2013)	87.9%	1,178	93.4% (91.0%–96.0%)	2.8
Northeast/Mombin-Crochu (2013)	87.4%	1,184	87.0% (83.0%–91.0%)	6.0
Northeast/Ouanaminthe (2013)	114.3%	1,111	88.6% (86.0%–92.0%)	4.1
Nippes/Petite-Rivière-de-Nippes (2013)	89.9%	1,033	91.1% (87.0%–95.0%)	4.8
**Average/total**	**95.4%**	**10,907**		

^1^Design effect is a measure of the ratio of true sampling variance under a specific design over the variance that would have resulted if the sample had been drawn as a simple unclustered random sample. The higher the design effect, the lower the precision of the estimates.

KAP questions were added to the coverage surveys in both 2012 and 2013 (Table 3). Results showed that it was easy for people to reach treatment posts (96% of respondents in 2012 and 82% in 2013 reported that the treatment post was “easily accessible” or “not too far”) and that awareness levels on the program were high (95% of respondents in 2012 and 89% in 2013 reported knowing the MDA was going to occur). While knowledge of signs and symptoms of LF was good (85% of respondents in 2012 and 59% in 2013 correctly reported at least one sign or symptom of LF), knowledge on prevention methods was low (27% of respondents in 2012 and 47% in 2013 correctly reported at least one method to prevent infection). A similar study in Léogâne following a single MDA, conducted by MSPP with support from CDC, had found no significant association between knowledge of disease and treatment [25].

**Table 3:** Variables associated with treatment outcomes.

Predictor variable		% (n) persons who TOOK TREATMENT			
	Persons responding positively to predictor % (n)	Of those with POSITIVE response to predictor variable	Of those with NEGATIVE responses to predictor variable	Adjusted odds ratio^4^	Adjusted p-value^5^ (t-test)
Knew MDA was going to happen in advance					
2012^6^	95.3% (1,182)	97.9% (1,157)	88.1% (52)	5.8	0.0005 (3.89)
2013^7^	89.6% (3,205)	90.1% (2,888)	45.5% (153)	12.1	<0.001 (9.7)
Accurately report signs and symptoms of LF^1^					
2012^6^	84.6% (1,050)	97.8% (1,027)	95.3% (182)	1	0.06 (1.93)
2013^7^	59.6% (2,133)	88.9% (1,896)	79.9% (1,154)	1.8	0.002 (3.47)
Accurately report prevention strategies for LF^2^					
2012^6^	27.9% (346)	97.7% (338)	97.3% (871)	1	0.10 (1.71)
2013^7^	47.5% (1,699)	90.8% (1,543)	80.2% (1,507)	2.7	<0.001 (5.6)
Report easy access to treatment at posts^3^					
2012^6^	96.2% (1,194)	98.8% (1,180)	61.7% (29)	30.0	<0.001 (5.4)
2013^7^	82.3% (2,943)	96.7% (2,845)	32.3% (205)	56.2	<0.001 (13.9)

^1^Responding “*leg swelling/elephantiasis*,” OR “*hydrocele*,” OR “*chyluria*,” OR *“unable to work or cannot work as much as before”* to the question *“What are the signs and symptoms of LF*?*”*

^2^Responding “*use bed nets*,” OR “*avoid mosquito bites*,” OR “*take LF treatment*” to the question “*How is LF prevented*?”

^3^Responding *“close”* OR *“not too far”* to the question *“What would you say about the distance of the distribution post from your house*?*”* AND *“yes”* to the question *“Was the time the distribution post was open good for you*?*”* AND *“no”* to the question *“Did you ever go to a distribution post for pills and find that no one was there*?*”*

^4^ The odds ratio, adjusted for age and gender, compares the odds of taking treatment for those responding positively to predictor variables with the odds of taking treatment for those who responded negatively. For example, the odds of taking treatment, for those who knew the MDA was going to happen compared with those who did not know.

^5^ P-value, adjusted for age and gender, based on t-test from logistic regression analysis.

^6^ 2012 survey sample size = 1,241 with completed KAP questions.

^7^2013 survey sample size = 3,577 with completed KAP questions.

The factor most strongly associated with taking the treatment was access, with adjusted odds ratios of 30 and 56.2 for 2012 and 2013 respectively, p <0.001 (t-test). Caution should be exercised when interpreting the odds ratios not to overemphasize the significance of such high odds ratios. If an outcome is rare, odds ratios and relative risk would be roughly equivalent. Here, since the majority of the surveyed took the drug, the odds ratio and relative risk are different. The unadjusted relative risk is 2. Awareness of the program was also significantly associated with taking the drugs (odds ratios of 5.8 and 12.1 for 2012 and 2013 respectively, p <0.001 [t-test]). The association between knowledge of disease and taking treatment was inconclusive—knowledge was not associated with taking the treatment in 2012 but was significantly associated in 2013 –odds ratio of 1.8 (p = 0.002) for persons who could reports signs and symptoms and odds ratio of 2.7 (p <0.001) for ability to report prevention strategies.

### Impact of the Program on *W. bancrofti* Infection Rates

Program impact is first measured at sentinel and spot-check sites—these are conducted at midterm after two to three MDA rounds and again before proceeding to conduct the more extensive TAS, following a minimum of five MDA rounds. Once a commune has completed a minimum of five rounds of MDA, with epidemiological coverage >65% and where sentinel site *W*. *bancrofti* antigen levels are below 2%, TAS should be implemented to determine whether MDA can be stopped [24]. For these TAS, communes may be grouped into larger evaluation units (EUs) based on similar epidemiologic characteristics. Results are reported for areas supported by ENVISION only, where the program was implemented as described in the methods section.

**Fig 4:**
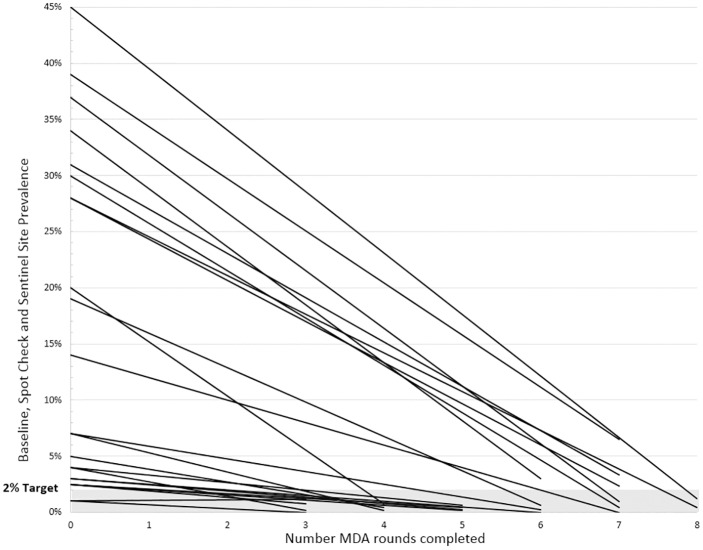
Decline in LF antigen level from start of program until 2014.

In 2014–2015, sentinel and spot-check surveys were conducted in 23 communes as part of either midterm or pre-TAS surveys (Table 4). In all communes, there was a marked decrease in infection prevalence as compared with initial mapping results. Results were below the threshold of 2% antigenemia in 19 of the 23 sites (Fig 4); 7 of those sites (conducting midterm assessments) had completed only three or four rounds of MDA, and all 7 had already reached the 2% threshold. MDA was continued in these areas based on current global guidelines to conduct a minimum of five rounds.

**Table 4:** Sentinel sites and spot checks data.

Commune (Department)	Number of consecutive MDA rounds conducted prior to assessment	Baseline ICT prevalence FY 01	Sentinel site ICT prevalence FY 14/15	Number of persons tested	District-level reported epi. coverage by FY					
					‘09	‘10	‘11	‘12	‘13	‘14
Les Cayes (South)	3	4.0%	0.19%	515	-	-	88%	74%	88%	85%
Port-à-Piment (South)	3	2.5%	0.77%	517	-	-	79%	91%	94%	96%
Abricots (Grand’Anse)	3	1.0%	0.00%	455	-	-	87%	79%	70%	81%
Pestel (Grand’Anse)	3	1.0%	1.15%	520	-	-	81%	77%	94%	90%
Caracol (Northeast)	4	20.0%	0.77%	517	-	104%	97%	117%	90%	101%
Sainte-Suzanne (Northeast)	4	7.0%	0.19%	532	-	79%	73%	66%	74%	73%
Trou-du-Nord (Northeast)	4	5.0%	0.4%	510	-	91%	90%	87%	103%	141%
Jacmel (Southeast)	5	2.5%	0.40%	500	104%	87%	69%	80%	84%	S
Anse-à-Pitres (Southeast)	5	2.5%	0.19%	527	91%	70%	69%	72%	68%	S
Anse-à-Veau (Nippes)	5	4.0%	0.60%	510	90%	89%	94%	109%	88%	S
L’Asile (Nippes)	5	3.0%	0.20%	505	85%	84%	79%	83%	88%	S
Anse-à-Foleur (Northwest)	6	3.0%	0.00%	517	80%	104%	84%	88%	86%	75%
Chansolme (Northwest)	6	7.0%	0.20%	504	151%	151%	104%	87%	85%	76%
Port-de-Paix (Northwest)	6	34.0%	**3.00%**	502	110%	96%	100%	96%	95%	95%
Limbé (North)	6	19.0%	0.60%	510	N/A	104%	108%	112%	101%	79%
Dondon (North)	7	14.0%	0.00%	510	N/A	101%	105%	104%	111%	104%
Milot (North)	7	31.0%	**3.35%**	507	N/A	83%	99%	89%	91%	85%
Limonade (North)	7	37.0%	0.98%	510	N/A	95%	109%	97%	127%	91%
Quartier-Morin (North)	7	39.0%	**6.50%**	510	N/A	90%	124%	96%	103%	100%
Plaisance (North)	7	30.0%	0.40%	510	66%	77%	104%	93%	90%	90%
Acul-du-Nord (North)	7	28.0%	**2.35%**	510	N/A	98%	98%	106%	83%	91%
Plaine-du-Nord (North)	8	45.0%	1.20%	502	N/A	100%	124%	89%	102%	99%
Cap-Haïtien (North)	8	28.0%	0.40%	510	N/A	109%	99%	89%	81%	85%

S = surveillance phase.

FY, fiscal year; ICT, immunochromatographic test.

On the other hand, four sites had large reductions but failed to drop below the critical threshold, even after six or seven rounds (Table 4). The principal difference between these two groups is that the 19 sites where infection rates were reported as below the 2% threshold typically had lower prevalence rates at baseline; despite not meeting the criteria to stop MDA, the substantial decrease in prevalence (i.e., ranging from 28% to 39% at baseline to between 2.3% and 6.5% pre-TAS) among those four sites is viewed as a programmatic success (Table 4).

By mid-2015, TAS had been implemented in a total of 13 EUs, 10 of which achieved the prevalence threshold required to stop MDA. These 10 EUs are made up of a total of 44 communes in which, according to WHO guidelines, MDA can now be stopped, while post-MDA surveillance will continue for an additional four to six years. In the other three EUs (made up of three communes), MDA will continue for a minimum of two more years.

For STH, a national survey was conducted in 2013 to determine the STH infection prevalence in school children (aged from 6 to 16 years old), at least five years after the program of annual MDA with ALB+DEC had begun. This survey showed that STH prevalence had decreased in 9 of 10 departments, with STH prevalence at or below 10% in 3 departments and at or below 20% in another 6 departments. In the tenth department (Grand’Anse), which had an initial prevalence of 72% when surveyed in 2001, the decline was not as pronounced as in the other nine, and the prevalence of infection remained at 55%[26]. Although these results are encouraging and show significant progress in addressing the problems caused by STH in Haiti, they also highlight the question of how progress will continue after support for annual MDA targeting LF ceases.

**Fig 5:**
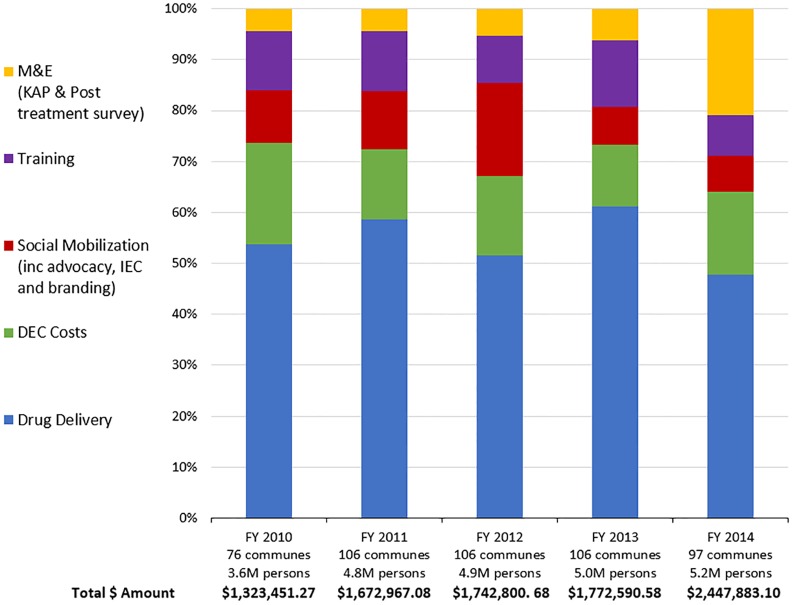
Project costs (FY 2010–2014).

Program costs. Detailed program cost assessments have been carried out for those communes where MSPP activities have been supported by ENVISION. Although relative costs for some of the program activities did vary slightly from year to year, drug delivery costs always predominated, especially when including social mobilization costs (Fig 5). Because ALB is provided by GlaxoSmithKline at no cost to the program, and since the DEC is quite inexpensive, the cost of medicines (i.e., the sine qua non of MDA strategies) to the national program remains relatively low.

Between 2010 and 2013, the per person treated cost of the program—characterized predominantly by MDA—has remained steady (US$0.35–0.37). However, in 2014 an increase in total budget was observed as the requirement for more comprehensive M&E efforts, including TAS surveys, became necessary.

## Discussion

By 2012, Haiti’s NTD program had reached national scale, with consistently good epidemiological coverage reported for MDA. Following TAS results that confirmed LF infection was below the targeted transmission thresholds, MDA could be stopped, resulting in a scaling down of geographical coverage. By 2015, 45 communes– 44 of which were in ENVISION-supported areas—had stopped MDA. Such results add to the growing body of global evidence supporting the feasibility and effectiveness of implementing WHO’s strategy to eliminate LF via once-yearly MDA for at least five years with a minimum of 65% epidemiological coverage [27].

**Fig 6:**
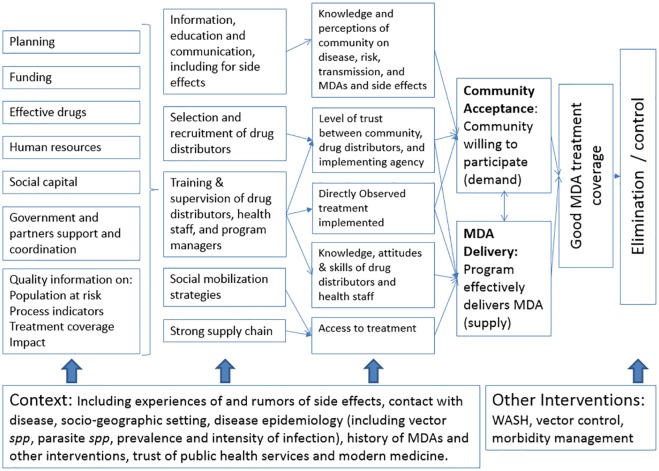
Theory of change for NTD programs.

To achieve these results, it is essential that MDA rounds are able to reach and sustain good coverage of the targeted population. In practical terms, the success of MDA programs hinges on two operationally critical elements: a population willing to accept the treatments being offered (community acceptance) and a health system able to deliver the treatments effectively to the community (MDA delivery). These in turn depend on a number of other essential factors, summarized in Fig 6 and described below.

### Community Acceptance

The likelihood of taking the offered treatment has been positively associated in a number of studies with the community’s knowledge and understanding of the disease, its transmission, MDA strategy, and possible side effects; the community’s perceived risk of getting the disease; and history of side effects experienced [28–33]. In Haiti, however, knowledge of disease signs and symptoms was generally low and not strongly associated with taking part in MDA. The most important factors in determining whether people took the treatment offered them were (1) knowing that the MDA was to take place and (2) the ease of access to treatment posts. It is also of note that treatment in Haiti’s NTD program is directly observed, a factor associated in other settings with increased likelihood of community participation [29,34–38].

Reluctance to take treatment due to fear of minor side effects was low, reflecting the importance placed on informing the community about expected side effects—achieved through emphasis during CDD training and through dissemination of IEC messages. The program also benefitted from the fact that there were no reported serious adverse events.

This program has been able to extensively leverage pre-existing relationships among ENVISION, the field MSPP personnel, and the community leaders—all of whom emphasize the involvement of the community, thereby building on the foundation of existing strong social capital. Other studies also support the idea that drug distributors who are known and respected by the community will have better success in treating the population [34].

### MDA Delivery

The factor most strongly associated with taking the drugs in this program was ready access to treatment (odds ratios of 30 and 56.2 for 2012 and 2013 respectively, p <0.001). The program’s attention to logistics—in terms of number and location of posts, as well as assurance that the posts are attended by well-trained and supported CDDs—accompanied by a strong supply chain that ensured the availability of treatments, were critical to the successful achievement of persistent high treatment coverage.

In light of the strong evidence presented on good coverage in MDA rounds obtained by the Haitian program, the excellent results of the assessment of impact on infection at sentinel, spot-check, and evaluation sites should not be surprising. LYMFASIM, a mathematical model predicting elimination of LF using an MDA approach, was developed based on a similar scenario in India, where *C*. *quinquefasciatus* is the vector and *W*. *bancrofti* the parasite, but where ivermectin was used instead of DEC. This model predicted that the number of rounds necessary to achieve elimination depends to a large extent on both coverage and the pre-program endemicity level [39]. This prediction was well corroborated in Haiti’s experience– 19 of the 23 sentinel sites tested in 2014–2015 reached below the critical 2% antigen prevalence threshold. The four sites that did not reach this threshold, despite at least six rounds of MDA with high epidemiological drug coverage (≥83%), had very high baseline antigen prevalence (28%–39%; Fig 4). Earlier studies in Léogâne, Haiti also reported slower declines—from 50% to only 14% following seven rounds of MDA [40].

As noted earlier, seven sentinel sites with initially much lower baseline prevalence had already reached below the <2% antigenemia threshold after only three to four rounds of MDA. These communes continued to implement another one to two rounds of MDA following current WHO guidelines. Researchers in Haiti’s La Tortue Island earlier reported a successful TAS survey and the stopping of MDA after just two rounds of treatment [16]. In the neighboring Dominican Republic, similar results—reduction of antigenemia to below threshold after three rounds of MDA—were found in the urban center of the country, again with low baseline prevalence and high program coverage [41]. There is potential for programmatic efficiencies if programs targeting LF elimination could be successful with fewer rounds of MDA than are now recommended; whether this would be sufficient to interrupt transmission will need to be determined by operational research.

### Challenges and Next Steps

In addition to completing efforts to eliminate LF, Haiti’s NTD program needs to sustain achievements of reduced STH burden. During recent years, the STH program has relied on the fact that ALB is administered to the whole population as part of the LF MDA. As the LF program progressively meets the criteria for stopping MDA, another platform will be needed for delivery of STH treatments. Haiti’s plan is to focus on school-based, STH-only MDA and to add water, sanitation, and hygiene activities.

In 2015, the InterAmerican Development Bank provided the MSPP with funds for a national deworming campaign. The InterAmerican Development Bank drew upon IMA’s expertise for support in training of drug distributors and working with department-level STH leads to formulate best-practice MDA strategies—illustrating the continued integration and collaboration between the two disease programs and the potential resources that have been developed in country and can be leveraged to ensure the sustainability of STH control activities.

As MDA activities are now reaching the stopping point, Haiti has started to turn its attention to morbidity control efforts for LF, building on hydrocele surgery efforts and lymphedema patient support groups that have been implemented in Léogâne [42]. The MSPP updated its national morbidity management and disability prevention (MMDP) strategic plan in early 2016, which includes rolling out the use of WHO’s new MMDP situation analysis tools to estimate the number of patients with hydrocele or lymphedema, identify platforms to support MMDP activities, identify strategies to mobilize patients, and identify the human resources and funding needs.

Finally, plans are also in place to work Hispaniola-wide on a malaria elimination plan, harnessing the momentum already gained for LF elimination and further increasing the probability of LF elimination though vector control [43].

### Conclusion

In summary, in this mostly rural Haitian setting, a very successful MDA campaign was launched in 2001 with sustained high coverage rates, resulting in steady progress toward LF elimination (with more MDA rounds being needed in areas with high baseline prevalence and fewer where prevalence was initially low). A strong foundation for ongoing STH control has also been established. The strategies utilized with proven success by the program are summarized in Box 1. This list is an important contribution to the development of the global evidence base on MDA best practices.

**Box 1. Summary of Best Practices utilized in Haiti's NTD Program**

- Careful selection of CDDs to distribute drugs—persons known, respected, and trusted within their communities
- Strong motivation of dug distributors resulting in less than 10% annual turnover
- Two days of initial training, followed by annual one-day refresher training—using a strengthened cascade system described above
- Low ratio of drug distributors to first-level supervisors (1:9) who in turn have clear supervision responsibilities
- Easy access to the drugs through numerous distribution posts set up within the community
- Strong enforcement in training and during supervision of a directly observed treatment policy
- An evidence-based, multi-channel communication approach, combined with high visibility of the MDA itself, resulting in high awareness among the community where the MDA is occurring
- Focus of key communication messages on where to get treatment and on side effects—strong knowledge on disease was not required.
- Community acceptance of minor side effects (headaches and nausea) as a result of emphasizing this information during drug distributor training (drug distributors also encourage community members to eat before the MDA)
- A strong drug supply chain as evidenced by good documentation, the lack of drug stock-outs, and very low drug wastage
- Post-MDA project review at all levels
- A strong M&E system put in place, following WHO guidelines
- A strong system to collect data from drug distributors on treatments given so that coverage rates can be followed and lead to action in lower performing areas
- The national government’s strong leadership and partnership both with the community and with international stakeholders

## Supporting Information

S1 Data 2012 and 2013 post treatment coverage survey KAP data. (XLSX)

S2 Data 2012 post treatment coverage survey data. (XLSX)

S3 Data 2012 post treatment coverage survey location data. (XLSX)

S4 Data 2013 post treatment coverage survey data. (XLSX)

S5 Data 2013 post treatment coverage survey location data. (XLSX)

S6 Data 2009–2014 program coverage data. (XLSX)

S1 Checklist STROBE Checklist. (DOCX)
